# Clustering of uveal melanoma: County wide analysis within Ohio

**DOI:** 10.1371/journal.pone.0290284

**Published:** 2023-08-18

**Authors:** Leanne M. Clevenger, Jacquelyn D. Wrenn, James Bena, Guneet Sodhi, Katherine Tullio, Arun D. Singh

**Affiliations:** 1 Cole Eye Institute, Cleveland Clinic, Cleveland, Ohio, United States of America; 2 Quantitative Health Sciences, Lerner Research Institute, Cleveland Clinic, Cleveland, Ohio, United States of America; 3 Cancer Health Analytics, Taussig Cancer Institute, Cleveland Clinic and Health Equity (Ohio), CareSource, Columbus, Ohio, United States of America; Eye Foundation Hospital / Eye Foundation Retina Institute, NIGERIA

## Abstract

**Purpose:**

To determine if a greater than expected number of cases (clustering) of uveal melanoma occurred within Ohio for any specific region or time period as compared to others.

**Design:**

Analysis of population database.

**Methods:**

Ohio Cancer Incidence Surveillance System (OCISS) database (2000–2019) was accessed for the diagnosis of uveal melanoma using the International Classification of Disease for Oncology codes: C69.3 (choroid), C69.4 (ciliary body and iris). Counties within Ohio were grouped by geographic regions (7) and socioeconomic variables. Age- and race-standardized incidence ratios (SIR) were calculated to determine temporal or geographic clustering.

**Results:**

Over the twenty-year period, the total number of uveal melanoma cases reported within Ohio were 1,617 with the overall age-adjusted annual incidence of 6.72 cases per million population (95% CI 6.30–7.16). There was an increase in the incidence of uveal melanoma over 20 years (p<0.001) across seven geographic regions, but no significant difference in incidence rates between the regions. There was no difference in incidence based on county classification by age composition (p = 0.14) or education level (p = 0.11). Counties with a low median household income (p<0.001), those classified as urban (p = 0.004), and those with a greater minority population (p = 0.004) had lower incidence. Less populated counties had a higher incidence of uveal melanoma (p<0.001).

**Conclusions:**

There is no evidence of geographic or temporal clustering of uveal melanoma within Ohio from 2000 to 2019.

## Introduction

Geographic clustering of cancer remains a source of persistent public safety concern [1, 2]. The Centers for Disease Control and Prevention (CDC) has defined a cancer cluster as “greater than expected number of cancer cases that occurs within a group of people in a geographic area over a defined period of time”[3]. Occurrence of cancer clustering is explored by calculating standardized incidence ratio (SIR) which is a ratio of the number of observed cases to the number of expected cases [3]. A confirmation of a cancer cluster can offer clues to the underlying etiology.

Considerable media attention has recently been given to proposed “clustering” or “geospatial accumulations” of patients diagnosed with uveal melanoma in Huntersville, North Carolina and Auburn, Alabama [4, 5]. The number of expected cases of uveal melanoma in a geographic region (with known population) can be readily estimated as the incidence (5.2 cases per million) [6] and prevalence (61.0–73.6 cases per million population) [7, 8]. of uveal melanoma are well known. However, calculation of SIR can be challenging as the geographic location and duration over which the incident cases were observed needs to be accounted for. Datasets such as those provided by the National Cancer Institute’s (NCI) Surveillance, Epidemiology, and End Results (SEER) program provide robust longitudinal data for all cancers in the US including uveal melanoma but cannot be probed for evidence of clustering as they do not provide geographic location (such as zip code or county) of the incident cases.

Through special permission and approval process from the Ohio Department of Health (ODH), we accessed and investigated 20 years of data (2000–2019) from the Ohio Cancer Incidence Surveillance System (OCISS) for potential temporal or geographic clustering of uveal melanoma. To our knowledge, this population dataset is unique as it includes residence (geographic location, by county within Ohio) of the patient at the time of uveal melanoma diagnosis.

## Methods

This study was performed following Institutional Review Board approval from the Ohio Department of Health and was conducted in full accordance with all applicable Ohio Department of Health’s Research Policies and Procedures. It adheres to guidelines and checkpoints established by the CDC regarding cancer cluster investigation, as well as HIPAA (Health Insurance Portability and Accountability Act of 1996) regulations [3]. This study investigated twenty years of data (2000–2019) from the OCISS database on cases of uveal melanoma within Ohio. Adult patients were included who were reported to be diagnosed with uveal melanoma to the OCISS database, identified using the International Classification of Disease for Oncology codes: C69.3 (choroid), C69.4 (ciliary body and iris), and C69.2 (retina). As “retinal melanoma” most likely represented miscoding of uveal melanoma, these cases were included in the analysis. Those patients with ocular melanoma coded as “site non specified” were excluded. Analyzed variables include county of residence at time of diagnosis, race/ethnicity, sex, age at diagnosis, and date of diagnosis.

### Dataset

Cancer incidence data used in these analyses were obtained from the Ohio Cancer Incidence Surveillance System (OCISS), Ohio Department of Health (ODH), a cancer registry partially supported by the National Program of Cancer Registries at the CDC through Cooperative Agreement Number NU58DP006284. Use of these data does not imply that ODH or CDC agrees or disagrees with the analyses, interpretation or conclusions in this report.

In accordance with Ohio law, each physician, dentist, hospital or person providing diagnostic or treatment services to patients with cancer must report each case of cancer to the OCISS at the ODH within six months of date of diagnosis and/or first contact with the facility [9]. Cancer reporting is performed electronically and adheres to standards for data collection as required by the CDC’s National Program of Cancer Registries (NPCR), as well as standards from the North American Association of Central Cancer Registries (NAACCR).

### Time periods

To evaluate for temporal clustering of uveal melanoma, the data was broken into four equal time periods: 2000–2004, 2005–2009, 2010–2014, and 2015–2019. Five-year intervals were selected owing to overall low incidence of uveal melanoma by county each year. Relative changes in population were calculated from year 2000.

### Geographic regions and county classification

The 88 counties within the state of Ohio were classified by geographic regions (7 regions as determined by the Ohio auditor- Northwest, Northeast, East, Central, West, Southwest, and Southeast) [10], population composition and socio-economic indicators that included: rural versus urban counties (USDA-rural or urban) [11, 12], total population (<24000, 24001–50000, 50001–90000, 90001–115000, 115001–300000, 300001–800000, and >800001) [13], age distribution of county population (youngest third, middle third, and oldest third) [14], minority (non-white) population (%) within county (<5%, 5–10%, 10–15%, 15–25%, and >25%) [13], education level (county population with bachelor’s degree) were divided into thirds (bottom third, middle third, and top third) [15], and by median household income within the county ($44000 or less, $44000-$56000, $57000-$68000, $69000-$80000, $81,000 or more) [16].

### Statistical analysis

Raw incidence data was collapsed into 5-year increments. Population data from the census at the county level was used to calculate rates. 2000 Census data was used for 2000–2004, 2010 Census data was used for 2005–2014, and 2020 data was used for 2015–2019. Direct standardization was performed by age group (18–64, 65+) and race (White vs. Other) to the 2010 Census levels for both age and race. To model incidence rates over time, Poisson regression models were used. When comparing classification measures, these models included a time by measure interaction to assess whether relationships were consistent over time. When the interaction was significant, separate comparisons of the measures were performed by time period. To evaluate associations among variables used to classify regions, Fisher exact tests, Wilcoxon rank sum tests, and Spearman correlations were fit. Plots of the rates by county as well as changes in population and rates over time were created using R software (version 4.0). All other analyses were performed using SAS software (version 9.4).

## Results

### Data quality measures

The OCISS conducted by state of Ohio typically meets the criteria for the Advanced National Data Quality Standard as described by the NPCR: (1) Data are 90% complete based on observed-to-expected cases computed by the CDC, (2) there is a 2 per 1,000 or fewer unresolved duplicate rate, (3) the maximum percent missing critical data elements are 3% for age, sex, and county, and 5% for race, (4) 97% pass a CDC-prescribed set of standard edits [17]. OCISS also meets the highest NAACCR standard for complete, accurate, and timely data to calculate standard incidence statistics for the year reviewed. This includes 95% case ascertainment or better, 3% or fewer death-certificate-only cases, fewer than 0.1% duplicate case reports, 100% error-free data variables used to crease incidence statistics by cancer type, sex, race, age, and county, less than 2% of cases missing information on age, sex, and county, less than 3% of cases missing race information, and submission to the NAACCR for evaluation within 23 months of the close of the diagnosis year under review [18].

### Population in Ohio

**Fig 1:**
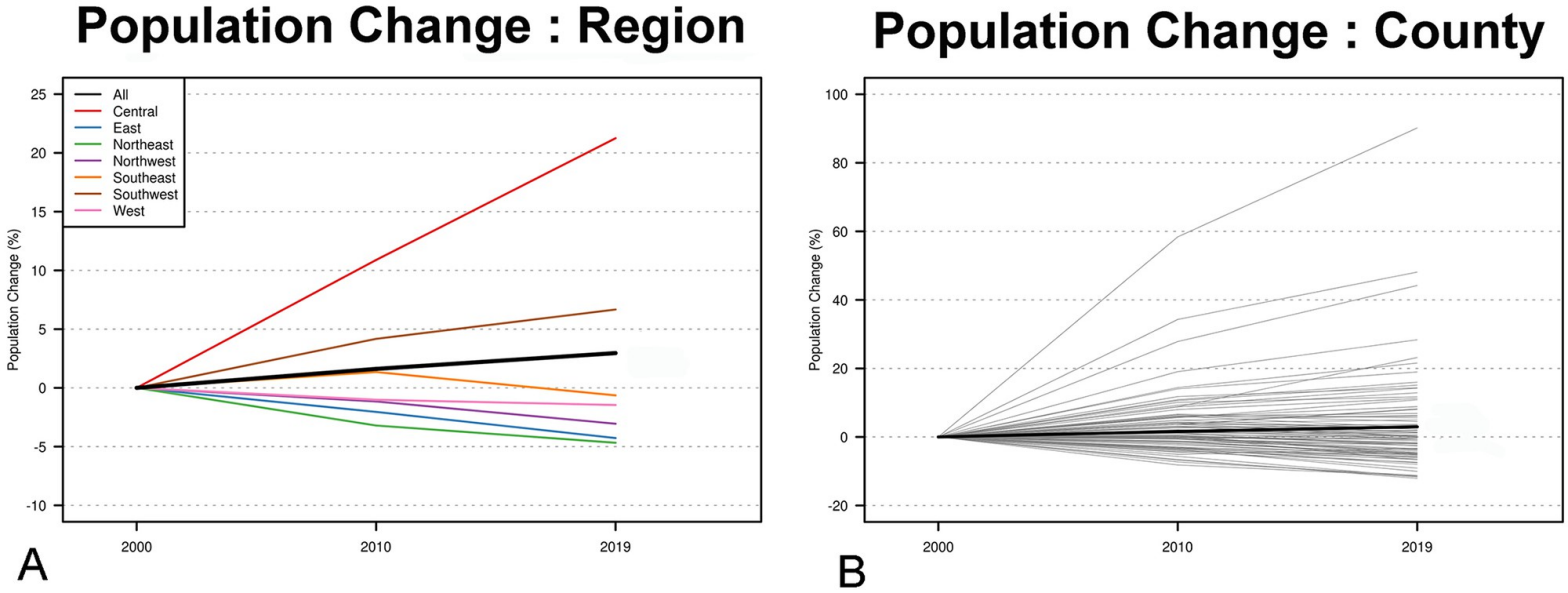
Uveal melanoma in Ohio. Population change by county (A), region (B) and overall.

Over the twenty-year period analyzed, there was insignificant increase in overall population (2.96%) between 2000 and 2020 (Fig 1).

### Incidence of uveal melanoma

Over the corresponding twenty-year period, the total number of uveal melanoma cases reported was 1,617. Overall, age-adjusted annual incidence of uveal melanoma within Ohio was 6.72 cases per million population (95% CI 6.30–7.16). The race adjusted rate was 6.36 (5.99, 6.76). The incidence gradually increased across each 5-year time period, which was statistically significant (p<0.001) (Table 1).

**Table 1:** Incidence of uveal melanoma in Ohio. Age standardized rates by year.

Year	Number of cases	Rate per million (95% CI)	Rate Ratio (95% CI)	Overall p-value
Overall	1617	6.72 (6.30, 7.16)		
2000–2004	289	5.17(4.59,5.83)		<0.001
2005–2009	364	6.16(5.49,6.90)	1.19(1.01,1.40)	
2010–2014	430	7.27(6.55,8.06)	1.40(1.23,1.60)	
2015–2019	534	8.20(7.41,9.08)	1.59(1.36,1.84)	

Poisson Regression Model Estimates. CI = Confidence Interval

Counties were evaluated by geographic region and population composition (Table 2). There was no region in Ohio with a significantly different incidence of uveal melanoma (p = 0.51). Those counties classified as rural had a higher incidence than those classified as urban (7.77 per million vs 6.47 per million, p = 0.004). Less populated counties had a higher incidence than more populated counties (p<0.001).

**Table 2:** Age-standardized incidence of uveal melanoma in Ohio counties (88): Geographic regions, population composition, and socioeconomic indicators.

Classification	Groups	Number (%)	Rate per million (95% CI)	Rate Ratio (95% CI)	Overall P-value
Overall		88 (100)	6.72 (6.30, 7.16)		
Region	East	8 (9.1)	6.23 (5.58,6.96)		0.51
	Central	15 (17.0)	6.81 (5.61,8.28)	1.09 (0.87,1.37)	
	Southwest	13 (14.8)	6.75 (5.90,7.73)	1.08 (0.91,1.29)	
	West	14 (15.9)	6.60 (6.08,7.16)	1.06 (0.92,1.21)	
	Northwest	15 (17.0)	6.85 (5.75,8.17)	1.10 (0.89,1.35)	
	Northeast	6 (6.8)	6.50 (5.60,7.55)	1.04 (0.87,1.26)	
	Southeast	17 (19.3)	8.26 (6.61,10.33)	1.33 (1.03,1.70)	
Type	Urban	40 (45.5)	6.47 (6.07,6.90)		***0*.*004***
	Rural	48 (54.5)	7.77 (6.97,8.65)	1.20 (1.06,1.36)	
Population (x1000 Numbers)	> 800	3 (3.4)	5.84 (5.73,5.95)		***< 0*.*001***
	300–800	6 (6.8)	6.22 (5.82,6.65)	1.06 (0.99,1.14)	
	115–300	15 (17.0)	7.51 (6.75,8.36)	1.29 (1.15,1.43)	
	90–115	6 (6.8)	7.05 (6.37,7.80)	1.21 (1.09,1.34)	
	50–90	19 (21.6)	7.32 (6.41,8.36)	1.25 (1.10,1.43)	
	24–50	31 (35.2)	7.23 (5.93,8.82)	1.24 (1.01,1.51)	
	< 24	8 (9.1)	10.94 (7.51,15.93)	1.87 (1.29,2.73)	
Population (Age Composition)	Youngest	29 (33.0)	6.70 (6.05,7.43)		0.14
	Middle	28 (31.8)	6.91 (6.37,7.49)	1.03 (0.90,1.18)	
	Oldest	31 (35.2)	7.78 (6.93,8.73)	1.16 (0.99,1.35)	
Population (%Minority)	15+%	13 (14.8)	6.40 (5.97,6.86)		***0*.*004***
	10–15%	14 (15.9)	7.62 (6.53,8.89)	1.19 (1.01,1.41)	
	5–10%	32 (36.4)	7.51 (6.79,8.30)	1.17 (1.04,1.33)	
	<5%	29 (33.0)	8.21 (7.03,9.58)	1.28 (1.08,1.52)	
Population (Education)	High	29 (33.0)	6.52 (6.07,7.02)		0.11
	Middle	29 (33.0)	6.95 (6.25,7.72)	1.06 (0.94,1.21)	
	Low	30 (34.1)	7.68 (6.69,8.81)	1.18 (1.01,1.37)	
Population (Median Household Income X 1000$)	<44	12 (13.6)	6.69 (6.11,7.33)		***< 0*.*001***
	44–56	50 (56.8)	6.34 (5.87,6.84)	0.95 (0.84,1.07)	
	57–68	21 (23.9)	8.38 (7.29,9.63)	1.25 (1.06,1.48)	
	69–80	2 (2.3)	7.96 (6.62,9.58)	1.19 (0.97,1.46)	
	>80	3 (3.4)	10.33 (10.33,10.33)	1.54 (1.41,1.69)	

### Temporal pattern

**Fig 2:**
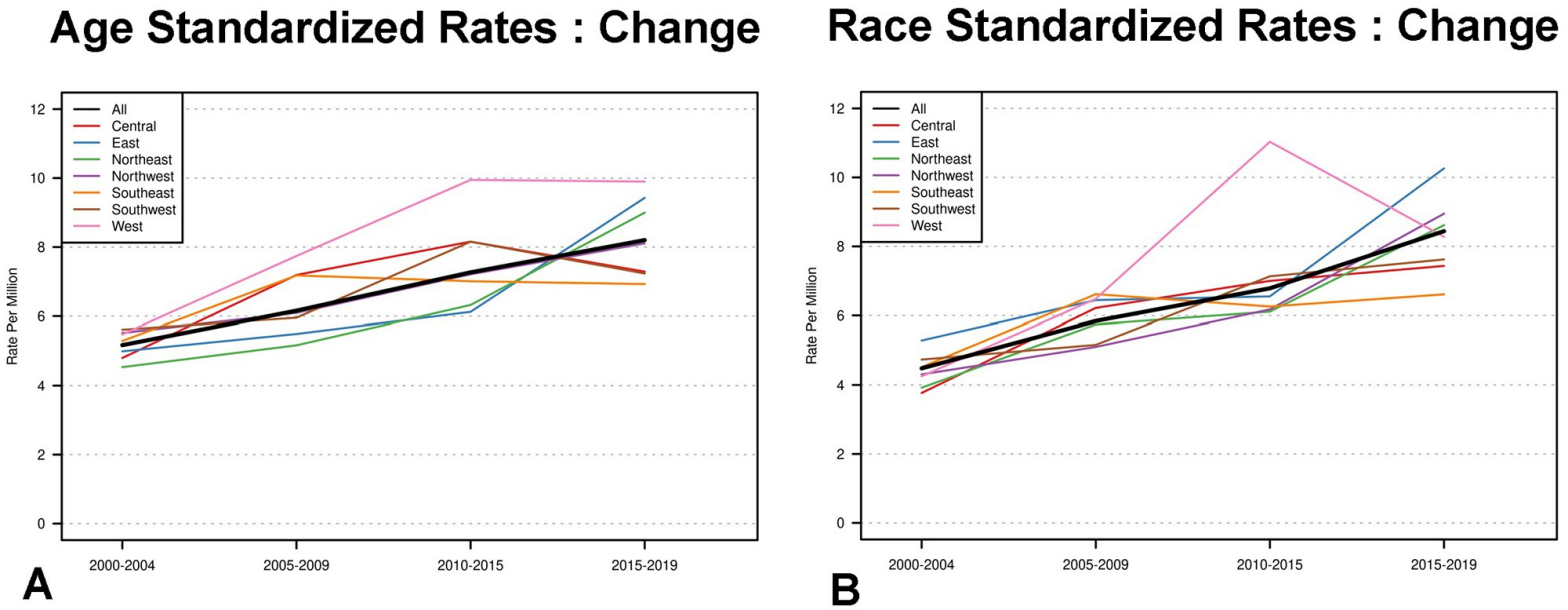
Uveal melanoma in Ohio. Age standardized (A) and race standardized incidence rate (B) change over time by region and overall (solid black).

There was increase in the incidence of uveal melanoma across each 5-year time period (p<0.001), which grew from 5.17 cases per million in 2000–2004 (95% CI 4.59, 5.83) to 8.20 cases per million from 2015–2019 (CI 7.41–9.08) (Table 1). This was also reflected as a gradual increase in overall age and race adjusted incidence rates throughout the 20-year period (Fig 2). There was no significant difference in incidence rates between the regions.

### Socioeconomic variables

Association of socioeconomic indications with incidence were also evaluated (Table 2). There was no difference in the incidence by education level (p = 0.11). Counties with a median household income of $44,000–56,000 had a lower incidence of uveal melanoma than those with lower or higher incomes (p = <0.001).

### Association between rural status and socioeconomic/other variables

**Fig 3:**
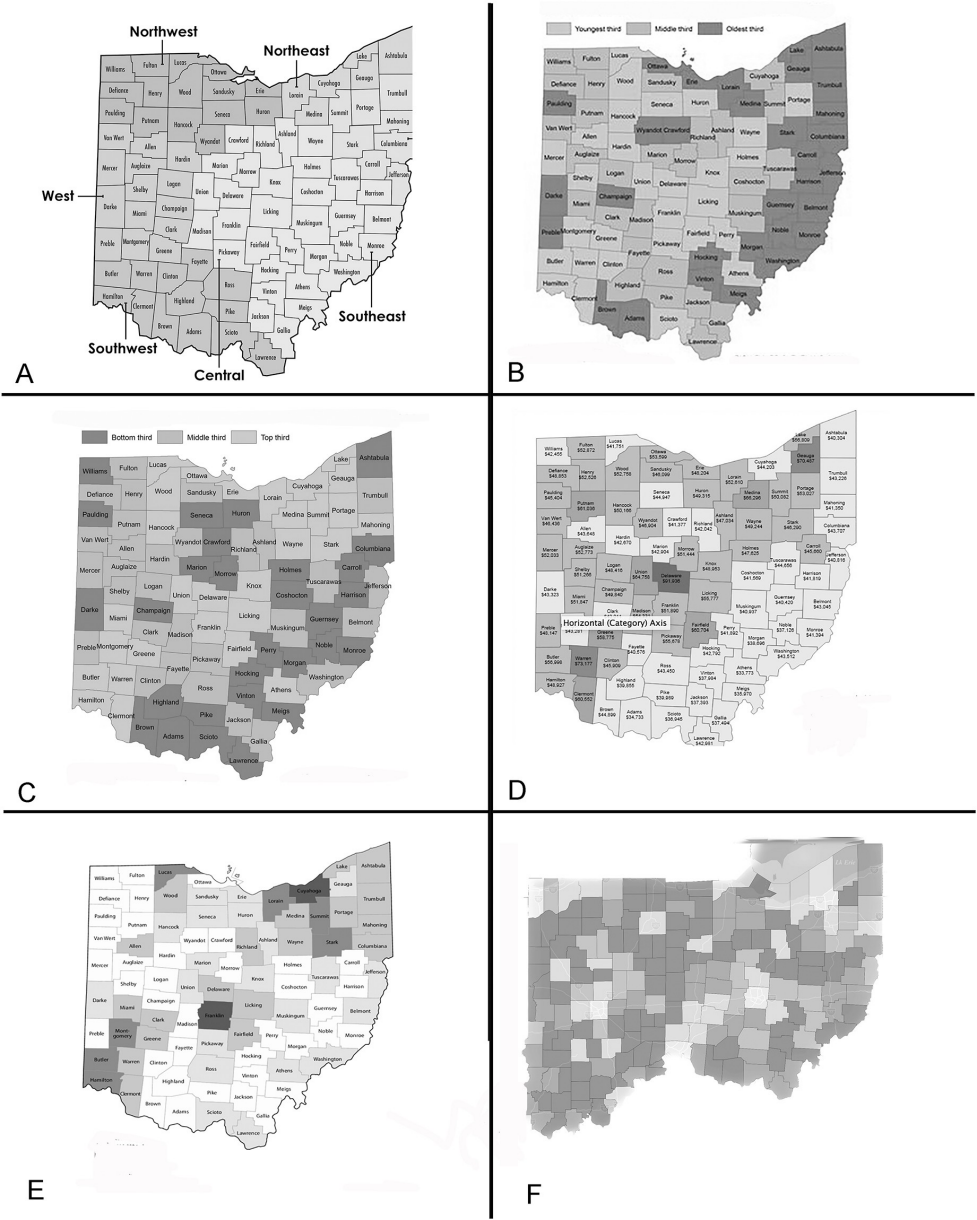
The 88 counties within the state of Ohio classified by 7 geographic regions (A) [10], age distribution of county population (B, youngest third, middle third, and oldest third) [14], education level (C, bottom third, middle third, and top third) [15], by median household income (D, $44000 or less, $44000-$56000, $57000-$68000, $69000-$80000, $81,000 or more) [16], total population (E, <24000, 24001–50000, 50001–90000, 90001–115000, 115001–300000, 300001–800000, and >800001) [13], and population composition (only-white) population (%) within county (F, <5%, 5–10%, 10–15%, 15–25%, and >25%) [13].

Counties that were classified as rural were more likely to be in the northwest or southeast region, and have lower education, income, population, and minority status (Table 3). In addition, weak correlations between income and population (r = 0.22), and income and minority percentage (r = 0.22) were observed, while a stronger association between population and minority percentage (r = 0.66) was also observed (Fig 3).

**Table 3:** Association between rural status and socioeconomic/other variables.

Factor	Rural (N = 48)		Urban (N = 40)		p-value
	N	Statistics	N	Statistics	
Region	48		40		***0*.*003*** ^c^
northwest		10 (20.8)		5 (12.5)	
northeast		1 (2.1)		5 (12.5)	
east		1 (2.1)		7 (17.5)	
central		6 (12.5)		9 (22.5)	
west		8 (16.7)		6 (15.0)	
southwest		7 (14.6)		6 (15.0)	
southeast		15 (31.3)		2 (5.0)	
Age	48		40		0.64^b^
Youngest 3rd		13 (27.1)		16 (40.0)	
Middle 3rd		19 (39.6)		9 (22.5)	
Oldest 3rd		16 (33.3)		15 (37.5)	
Education	48		40		***<0*.*001*** ^b^
Bottom 3rd		26 (54.2)		4 (10.0)	
Middle 3rd		18 (37.5)		11 (27.5)	
Top 3rd		4 (8.3)		25 (62.5)	
Income	48		40		***0*.*003*** ^b^
<44k		29 (60.4)		13 (32.5)	
44k-56k		18 (37.5)		19 (47.5)	
57k-68k		1 (2.1)		5 (12.5)	
69k-80k		0 (0.00)		3 (7.5)	
Population	48		40		***<0*.*001*** ^b^
<24,000		8 (16.7)		0 (0.00)	
24,001–50,000		24 (50.0)		7 (17.5)	
50,001–90,000		12 (25.0)		7 (17.5)	
90,001–115,000		3 (6.3)		3 (7.5)	
115,001–300,000		1 (2.1)		14 (35.0)	
300,001–800,000		0 (0.00)		9 (22.5)	
>800,000		0 (0.00)		0 (0.00)	
Minority	48		40		***<0*.*001*** ^b^
<5%		22 (45.8)		7 (17.5)	
5–10%		22 (45.8)		10 (25.0)	
10–15%		4 (8.3)		10 (25.0)	
15–25%		0 (0.00)		13 (32.5)	

Statistics presented as N (column %).

p-values:

^b^ = Wilcoxon Rank Sum test,

^c^ = Fisher’s Exact test

## Discussion

The OCISS database analyzed in this study is certified to be high quality as it meets the highest NAACCR standard in accurate reporting of cancer data, as well as the Advanced National Data Quality Standard as described by the NPCR [17, 18]. Although the OCISS database has never before been utilized in the investigation of uveal melanoma within Ohio, other high-quality dataset derived from state based cancer reporting systems have yielded incidence estimate (4.9 cases per million population) [19] which is comparable to those reported by the SEER program (5.2 per million, NCI based national database) [6]. While SEER dataset allows for accurate reporting of incidence of a rare disease, the database cannot be used to probe for evidence of clustering because the specific geographic location of the patient cannot be accessed.

Limited attempts have been made to evaluate for clustering of uveal melanoma. Davidorf and Knupp (1979) evaluated 698 patients who had enucleations for primary choroidal melanoma over an 11-year time period and found no evidence for temporal or geographic clustering of these cases by county within Ohio [20]. Their dataset is likely incomplete as it relied solely upon hospital reporting of enucleated cases. Albert et al. (1980) investigated clustering of patients with choroidal melanoma in a single population of chemical workers in West Virginia through examination of active plant employees and a control population of workers from the surrounding area [21]. Five cases of choroidal melanoma were found to have occurred from 1952 through 1978, which was determined to represent a statistically significantly greater than expected cases of choroidal melanoma [21]. No likely causal environmental exposure was later identified. Orloff et al. (2020) evaluated the unique geospatial accumulations of patients with uveal melanoma in North Carolina, Alabama, and New York. Standardized incidence ratios were determined for the states or counties involved, and no evidence for geographic or temporal clustering of uveal melanoma was found [22]. Such anecdotal case driven explorations for clustering are prone to ascertainment bias because the number of observed cases in the population at risk (from which incident cases are derived) cannot be accurately estimated [22].

In the present study, the overall annual incidence of uveal melanoma has increased over 20 years (Table 1) for which no obvious explanation can be offered given lack of data related to specific environmental or occupational exposure. However, statistically significant differences in incidence were found among several county groups. Those counties classified as rural, less populated, and those with less minority population had higher incidence reflecting correlation between the classification of counties as rural counties tend to be less populated and have less minority composition. Counties with median household income of $44,000 to $56,000 had a lower incidence (6.34 cases per million, 95% CI 5.87–6.84). Variations of incidence by counties can be explained by correlation between the rural status and greater likelihood of being in the northwest or southeast region and have lower education, income, population, and minority status (Table 3). The steady rise in incidence across Ohio, however, was not due to geographic or temporal clustering of uveal melanoma as both age and race standardized incidence rates did not differ significantly between any regions (Fig 2).

Various risk factors have been previously identified for uveal melanoma. Racial tendencies (lighter eye and skin color and the increased risk for developing uveal melanoma [OR 1.75 and 1.80], respectively) [23] and occupational exposures (welding and cooking [OR 2.05 and 1.81], respectively) are well known [23]. To better understand potential environmental and socioeconomic risk factors for uveal melanoma and other cancers, creation of unique patient identifiers that links the socioeconomic and occupational parameters to cancer registries such as the NAACCR and SEER database is required.

The limitations of our study is overall small number of cases despite our attempts to use 20 year state wide database which could have contributed to the lack of statistical power needed to detect a cluster [3]. Certain variables associated with a higher incidence (population less than 24,000) or lower incidence (income $44,000–56,000, rural, higher percent minority) of uveal melanoma can be explained on the overall low number of cases particularly in less populated counties and therefore, may not have any clinical relevance. In addition, influence of migration through its effects on total population, population composition within county, and socio-economic indicators could not be assessed.

In conclusion, we could not find evidence of geographic or temporal clustering of uveal melanoma within Ohio over the last 20 years.
